# Berberine regulates glucose metabolism in largemouth bass by modulating intestinal microbiota

**DOI:** 10.3389/fphys.2023.1147001

**Published:** 2023-03-09

**Authors:** Yun Xia, Hui-Ci Yang, Kai Zhang, Jing-Jing Tian, Zhi-Fei Li, Er-Meng Yu, Hong-Yan Li, Wang-Bao Gong, Wen-Ping Xie, Guang-Jun Wang, Jun Xie

**Affiliations:** ^1^ Key Laboratory of Tropical and Subtropical Fishery Resource Application and Cultivation, Ministry of Agriculture, Pearl River Fisheries Research Institute, Chinese Academy of Fishery Sciences, Guangzhou, China; ^2^ Hainan Fisheries Innovation Research Institute, Chinese Academy of Fishery Sciences, Sanya, China

**Keywords:** *Micropterus salmoides*, serum glucose, berberine, intestinal microbiota, glycolysis

## Abstract

This study examined the role of intestinal microbiota in berberine (BBR)-mediated glucose (GLU) metabolism regulation in largemouth bass. Four groups of largemouth bass (133.7 ± 1.43 g) were fed with control diet, BBR (1 g/kg feed) supplemented diet, antibiotic (ATB, 0.9 g/kg feed) supplemented diet and BBR + ATB (1g/kg feed +0.9 g/kg feed) supplemented diet for 50 days. BBR improved growth, decreased the hepatosomatic and visceral weight indices, significantly downregulated the serum total cholesterol and GLU levels, and significantly upregulated the serum total bile acid (TBA) levels. The hepatic hexokinase, pyruvate kinase, GLU-6-phosphatase and glutamic oxalacetic transaminase activities in the largemouth bass were significantly upregulated when compared with those in the control group. The ATB group exhibited significantly decreased final bodyweight, weight gain, specific growth rates and serum TBA levels, and significantly increased hepatosomatic and viscera weight indices, hepatic phosphoenolpyruvate carboxykinase, phosphofructokinase, and pyruvate carboxylase activities, and serum GLU levels. Meanwhile, the BBR + ATB group exhibited significantly decreased final weight, weight gain and specific growth rates, and TBA levels and significantly increased hepatosomatic and viscera weight indices and GLU levels. High-throughput sequencing revealed that compared with those in the control group, the Chao one index and Bacteroidota contents were significantly upregulated and the Firmicutes contents were downregulated in the BBR group. Additionally, the Shannon and Simpson indices and Bacteroidota levels were significantly downregulated, whereas the Firmicutes levels were significantly upregulated in ATB and BBR + ATB groups. The results of *in-vitro* culture of intestinal microbiota revealed that BBR significantly increased the number of culturable bacteria. The characteristic bacterium in the BBR group was *Enterobacter cloacae*. Biochemical identification analysis revealed that *E. cloacae* metabolizes carbohydrates. The size and degree of vacuolation of the hepatocytes in the control, ATB, and ATB + BBR groups were higher than those in the BBR group. Additionally, BBR decreased the number of nuclei at the edges and the distribution of lipids in the liver tissue. Collectively, BBR reduced the blood GLU level and improved GLU metabolism in largemouth bass. Comparative analysis of experiments with ATB and BBR supplementation revealed that BBR regulated GLU metabolism in largemouth bass by modulating intestinal microbiota.

## 1 Introduction

Largemouth bass (*Micropterus salmoides*; also known as California bass), a carnivorous fish belonging to the order Perciformes and the family Centrarchidae, is a freshwater fish native to North America. Additionally, largemouth bass has a high nutritional value with high protein and fat contents and strong disease resistance properties, which contribute to their high economic value. Furthermore, largemouth bass is a freshwater aquaculture species with development potential. Recently, the method for largemouth bass culture has changed from traditional feeding of chilled bait to feeding with feed. Carbohydrates in feeds are one of the main energy sources for animals and are inexpensive and widely available. The supplementation of feeds with the right proportion of carbohydrates can promote the growth of aquatic animals, save protein, and reduce feed costs. However, the supplementation of feed with high levels of carbohydrates can markedly increase the blood sugar content of fish, leading to metabolic disorders, liver hypertrophy and necrosis, reduction in fish feeding activity, growth inhibition, and death, especially in carnivorous fish, such as largemouth bass. Largemouth bass has a low capacity for sugar loading and removal (Ma et al., 2019) and can exhibit physiological growth if the feed sugar level is maintained at approximately 10% (Xu et al., 2016). However, increasing the feed sugar levels will lead to the development of a severe “hepatobiliary syndrome,” which is characterised by persistent hyperglycaemia, hepatomegaly, hepatic glycogen accumulation, slowed growth rates, and reduced feed efficiency, and can cause mass mortality in severe cases, in largemouth bass (Lin et al., 2018).

Berberine (BBR), the active ingredient of the Chinese herbal medicine *Coptis chinensis* Franch., is an isoquinoline alkaloid found in various medicinal plants. In clinical medicine, BBR has been widely used for the treatment of diabetes. Some studies have demonstrated significant regulatory effects of BBR on glucolipid metabolism (Li et al., 2019). The use of BBR as a functional feed additive in aquaculture can alleviate oxidative stress caused by high-fat feeds in blunt snout bream and exert regulatory effects on lipid metabolism (Lu et al., 2017; Zhou et al., 2019). The addition of BBR to feed significantly reduced serum glucose (GLU), total cholesterol (TC), and triglyceride (TG) levels in grass carp and largemouth bass (Pan et al., 2019; Xia et al., 2022a). Pharmacological studies have reported that the bioavailability of BBR is poor with a large proportion (>90%) retained in the intestine after oral administration, resulting in low blood levels in humans and mammals (Ma et al., 2013; Tan et al., 2013). Mammalian studies have demonstrated that BBR regulates host blood GLU levels by modulating intestinal microbiota (Wang et al., 2018; Guo et al., 2019). Previously, we reported that BBR intake altered the gut microbial composition of largemouth bass and hypothesised that the blood GLU-lowering effect of BBR in largemouth bass may be related to the regulation of intestinal microbiota composition (Xia et al., 2022b).

Based on the results of the previous study, which indicated that dietary supplementation with BBR at 1 g/kg was beneficial for promoting growth and health in largemouth bass (Xia et al., 2022b). BBR (1 g/kg feed) was supplemented to the feed of largemouth bass in this study. Largemouth bass was divided into the following four groups by adding BBR (1 g/kg feed) and/or compound antibiotics (ATBs; metronidazole (200 mg/kg feed), gentamicin sulfate (200 mg/kg feed), neomycin sulfate (200 mg/kg feed), ampicillin sodium (200 mg/kg feed) and vancomycin (100 mg/kg feed) (Jin, 2020) to the feed: control, BBR, ATB, and ATB + BBR groups. This study aimed to determine if BBR downregulated blood GLU in largemouth bass only by regulating intestinal flora. The direct effect of BBR on intestinal microbiota was also verified using *in-vitro* culture.

## 2 Materials and methods

All animal studies in this paper were performed according to the relevant national and international guidelines. All animal care and experimental procedures were approved by the Chinese Academy of Fishery Science of the Pearl River Fisheries Research Institute (LAEC-PRFRI-2022-03-01).

### 2.1 Experimental materials and experimental design

Largemouth bass was obtained from “Superior Bass No. Three”, which was bred by the Pearl River Fisheries Research Institute. The fish were domesticated and fed on a standardised diet for 2 weeks before the experiment. Before the start of the experiment, the fish were allowed to fast for 24 h and weighed after anesthetization with MS-222 (Sigma; 0.1 g/kg bodyweight). Largemouth bass (n = 360; initial weight = 133.7 ± 1.43 g) were randomly assigned to 12 cage nets (capacity: 280L, 30 fish per tank) in a concrete pond and classified into the following four dietary treatment groups (Table 1): control, fed on a standardised commercial diet; BBR, fed on BBR (1 g/kg feed, Solarbio)-supplemented feed; mixed ATB group, fed on mixed ATB (0.9 g/kg feed: metronidazole (200 mg/kg feed, Sangon), gentamicin sulfate (200 mg/kg feed, Solarbio), neomycin sulfate (200 mg/kg feed, Solarbio), ampicillin sodium (200 mg/kg feed, Solarbio) and vancomycin (100 mg/kg feed, Macklin))-supplemented feed; ATB + BBR group, fed on BBR (1 g/kg feed) and mixed ATB (0.9 g/kg feed)-supplemented feed. Largemouth bass in each dietary treatment group was randomly assigned to three cage nets. Feeding was performed twice a day (8:00–9:00 a.m.; 5:00–6:00 p.m.) with full feeding. All uneaten feed pellets were collected, dried, and weighed at 65°C. Aerated tap water served as the source of water for aquaculture. The experimental conditions were as follows: water temperature, 17C–23°C, dissolved oxygen, 6–9 mg/L; total phosphorus, 0.19 ± 0.07 mg/L; total nitrogen, 2.45 ± 0.39 mg/L; ammonia nitrogen, 0.23 ± 0.06 mg/L; nitrite nitrogen, 0.52 ± 0.08 mg/L; nitrate nitrogen, 0.56 ± 0.12 mg/L; pH, approximately 8.53; culture cycle, 50 days; photoperiod, natural.

**TABLE 1:** Ingredient and proximate composition of experimental diets (g).

Dietary ingredient (%)	Control	BBR	ATB	BBR + ATB
Fish meal (Peru; 67 cp%)	250	250	250	250
Fish meal (63 cp%)	220	220	220	220
Chicken meal (United States; 65 cp%)	140	140	140	140
Soy protein concentrate	95	95	95	95
Soybean meal (46 cp%)	40	40	40	40
Fermented soybean meal	50	50	50	50
High-gluten flour (14 cp%)	50	49	49.1	48.1
Cassava starch	50	50	50	50
Soybean oil	60	60	60	60
Largemouth Bass Premix 1 (2%)	20	20	20	20
l-Lysine hydrochloride (98%)	3	3	3	3
dl-Methionine (99%)	2	2	2	2
Ca(H_2_ PO_4_)_2_	15	15	15	15
Choline chloride (50%)	0.5	0.5	0.5	0.5
BBR (98%, Ruitaibio)	0	1	0	1
ATB	0	0	0.9	0.9
Total	1,000	1,000	1,000	1,000
Crude protein (%)	51.43	51.43	51.42	51.40
Crude lipid (%)	12.33	12.33	12.33	12.33
Calcium (%)	3.08	3.08	3.08	3.08
Phosphorus (%)	2.15	2.15	2.15	2.15
Lysine (%)	3.73	3.73	3.73	3.73
Methionine (%)	1.24	1.24	1.24	1.24

Note: BBR, berberine; ATB, antibiotics; ATBs, comprised metronidazole (200 mg/kg feed, Sangon), gentamicin sulfate (200 mg/kg feed, Solarbio), neomycin sulfate (200 mg/kg feed, Solarbio), ampicillin sodium (200 mg/kg feed, Solarbio), and vancomycin (100 mg/kg feed, Macklin).

### 2.2 Sample collection

At the end of the 50-day feeding period, largemouth bass was allowed to fast for 24 h before sampling. From each group, 12 fish were randomly selected and anesthetised with MS-222. The bodyweight and length of the fish were measured. The blood samples were quickly collected from the tail vein into Eppendorf tubes, incubated overnight at 4°C, and centrifuged at 4°C and 3,000 g for 10 min. The supernatant was transferred into new Eppendorf tubes and stored at −80°C for subsequent analysis. Specimens were dissected on a sterile bench and weighed. The visceral weight and hepatopancreas weight were recorded. Approximately 0.4 cm × 0.4 cm × 0.4 cm of the liver tissue mass from each fish was soaked in 4% formaldehyde solution and sectioned. The sections were subjected to haematoxylin and eosin (HE) staining and Oil Red O staining. Tissues, such as the remaining liver, midgut and hindgut, and all intestinal contents were collected, snap-frozen in liquid nitrogen, and stored at −80°C for subsequent testing and analysis.

Growth indicator calculation formulas:

$$WGR=\left(m_{2}–m_{1}\right)/m_{1}\times 100\%$$

$$SG=\left(\mathrm{Ln}\;m_{2}–\mathrm{Ln}\;m_{1}\right)/t\;\times \;100\%$$

$$Wv=m_{v}/m_{b}\times 100\%$$

$$W_{h}=m_{h}/m_{b}\times 100\%$$

where WGR is the weight gain rate (%), SG is the specific growth rate (%/d), Wv is the viscera weight index (%), W_h_ is the hepatosomatic index (%), m_1_ is the initial mean weight of fish (g), m_2_ is the final mean weight of fish (g), t is the number of feeding days, m_h_ is the final liver weight per fish (g), m_v_ is final viscera weight per fish (g), m_b_ is the final bodyweight per fish (g).

### 2.3 Measurement of serum and liver biochemical parameters

The serum levels of TC, total bile acid (TBA), GLU, TG, high-density lipoprotein-cholesterol (HDL-C), low-density lipoprotein-cholesterol (LDL-C), glutamic oxalacetic transaminase (GOT), alkaline phosphatase (AKP), acid phosphatase (ACP) and liver GOT were measured using Nanjing Jiancheng Biotechnic Institute kits. Enzyme-linked immunosorbent assay kits (Shanghai Enzyme-linked Biotechnology Co., Ltd., China) were used to determine the liver GOT, phosphoenolpyruvate carboxykinase (PEPCK), phosphofructokinase (PFK), hexokinase (HK), pyruvate kinase (PK), pyruvate carboxylase (PC), and GLU-6-phosphatase (G6P) activities in largemouth bass.

### 2.4 *In-vitro* culture of intestinal microorganisms

*In-vitro* culture of intestinal microorganisms of largemouth bass was performed according to a previously reported method with some modifications (Krajmalnik-Brown et al., 2012; Hanning and Diaz-Sanchez 2015; Liu et al., 2019; Aranda-Díaz et al., 2022). Briefly, the gut contents of six largemouth bass were collected, and the faecal slurry was prepared by mixing fresh faecal samples with autoclaved saline into a 10% (w/v) suspension. The faecal slurry was mixed with autoclaved basal nutrient growth medium and preincubated for 60 min in an anaerobic incubator (Shanghai Yuejin Medical Optical Instrument Factory, China) at 37°C under N_2_ gas. The basal nutrient medium (pH 7.0) composition was as follows: peptone (2 g/L), soybean peptone (3.0 g/L), yeast extract (2 g/L), beef meal (2.2 g/L), digested serum powder (13.5 g/L), beef liver extract (1.2 g/L), GLU (3.0 g/L), KH_2_PO_3_ (2.5 g/L), sodium chloride (3.0 g/L), soluble starch (5.0 g/L), L-cysteine (0.3 g/L), sodium thioglycolate (0.3 g/L), vitamin K1 (0.1%), haeme chloride (5 mg/ml), and distilled water.

Different concentrations of BBR (10 μL) and mixed ATB (10 μL) were added to the intestinal bacterial cultures (990 μL). Saline (10 μL) was used as a negative control (control). The preincubated intestinal microbes were divided into the following nine groups: control (saline), B1 (final concentration of BBR in the culture system = 10 μg/ml), B2 (final concentration of BBR in the culture system = 20 μg/ml), A1 (final concentration of mixed ATB in the culture system = 1 μg/ml), A2 (final concentration of mixed ATB in the culture system = 10 μg/ml), B1 + A1, B1 + A2, B2 + A1, and B2 + A2 groups. The groups were laid in a grid of three each and incubated in 6-well plates (Krajmalnik-Brown et al., 2012; Hanning and Diaz-Sanchez 2015; Aranda-Díaz et al., 2022) at 37°C for 24 h. Next, 100 μL of the bacterial suspension was diluted by a factor of 10^3^, 10^4^, and 10^5^. The diluted broth was spread on a solid medium and incubated anaerobically at 37°C for 24 h. Specific single colonies from each treatment group were picked for amplification and sequencing using the universal primers 27F and 1492R (Dees and Ghiorse 2001; Kim et al., 2011).

Additionally, the bacterial broth was subjected to low-speed centrifugation, and most of the medium was removed. The samples were rinsed 1–3 times with sterile phosphate-buffered saline (pH = 7.4). Finally, the collected microbial samples were placed in 1.5 ml EP tubes, sealed with sealing film, stored at −80°C, and subjected to DNA extraction for subsequent analysis.

### 2.5 Intestinal microbiota DNA extraction, amplification, and sequencing

Bacterial DNA was extracted from 200 mg of intestinal contents of each largemouth bass, as well as from isolated gut microbial culture broth using a bacterial DNA extraction kit (Omega Bio-Kit, Norcross, United States). The intestinal contents of largemouth bass were subjected to 16S rRNA gene (V3 + V4 region) sequencing using high-throughput sequencing technology (Novogene Co., Beijing, China). For Solexa polymerase chain reaction (PCR), a 20-µL reaction mixture comprising 5 µL of PCR purification product, 2.5 µL of 2 μM forward primers, 2.5 µL of 2 μM reverse primers, and 10 µL of 2× Q5 HF MM was prepared. The PCR conditions were as follows: 98°C for 30 s, followed by 10 cycles of 98°C for 10 s, 65°C for 30 s, and 72°C for 30 s, and 72°C for 5 min. PCR products were mixed with the same volume of 1× loading buffer and subjected to agarose gel electrophoresis using a 1.5% gel. Amplicons with a size of 400–450 bp were selected for the next step. PCR products were mixed at an equal density ratio and purified using the OMEGA Gel Extraction Kit (Thermo Fisher Scientific, Waltham, MA, United States). Fragments were sequenced after gel cutting and recovery. The sequence data are deposited in the NCBI Sequence Read Archive (SRA) database under the bioproject id PRJNA931008 (https://www.ncbi.nlm.nih.gov/sra/PRJNA931008).

### 2.6 Biochemical identification analysis of isolated cultures of characteristic bacteria

Based on the sequencing results of the isolated culture of the characteristic bacteria in Section 2.4, the identification kit (colorimetric method) for Enterobacteriaceae and other non-fastidious Gram-negative bacilli (API 20E kit, Shanghai bioMérieux Co., Ltd.) was used. The target bacteria were inoculated into the API 20E reagent strips according to the instructions, and the results were observed at 37°C for 18–24 h. The functions of bacteria were identified by colour reactions between bacteria and different substrates or colour changes after adding additional reagents. The identification results were obtained by checking the search table.

### 2.7 Data analysis

The experimental results are shown as the mean ± SEM. One-way ANOVA was used to statistically analyze the data for the differently treated fish groups with SPSS 25.0 software, and Duncan’s multiple range tests were applied as well to detect any anticipated significant differences between treated fish groups at a significant level of 95%. The Spearman correlation coefficient of intestinal bacteria and physiological and biochemical indexes was calculated in R 3.6.3 (*p* < 0.05: statistically significant within 95% confidence intervals).

## 3 Results

### 3.1 Largemouth bass growth indicators

As shown in Table 2, compared with that in the control group, the weight gain rate of largemouth bass was significantly higher in the BBR group (*p <* 0.05). Meanwhile, compared with those in the control group, the final body weight and specific growth rate were higher and the viscera weight and hepatosomatic indices were lower in the BBR group, but the changes were not significant (*p >* 0.05). Compared with that in the ATB and BBR + ATB groups, the final bodyweight, the weight gain and specific growth rates of largemouth bass were significantly higher in the BBR group (*p <* 0.05). Furthermore, compared with those in the control group, the final bodyweight and weight gain and specific growth rates were significantly lower (*p <* 0.05) and the viscera weight and hepatosomatic indices were significantly higher in the ATB group (*p* < 0.05). The final bodyweight and weight gain and specific growth rates were non-significantly downregulated in the BBR + ATB group (*p* > 0.05), whereas the viscera weight and hepatosomatic indices were non-significantly upregulated (*p* > 0.05).

**TABLE 2:** Effects of BBR and/or ATB on the growth performance of largemouth bass.

Groups	Control	BBR	ATB	BBR + ATB
Initial bodyweight (g)	131.56 ± 9.53	134.73 ± 10.37	135.7 ± 8.42	134.8 ± 11.30
Final body weight (g)	287.93 ± 6.42^bc^	306.05 ± 6.94^c^	257.46 ± 10.74^a^	278.1 ± 7.87^ab^
Relative weight gain (%)	118.87 ± 4.88^b^	127.15 ± 5.15^c^	89.72 ± 7.92^a^	106.31 ± 5.84^ab^
Specific growth rate (%/d)	1.56 ± 0.04^bc^	1.64 ± 0.05^c^	1.26 ± 0.09^a^	1.44 ± 0.06^b^
Viscera weight index	7.53 ± 0.20^ab^	7.08 ± 0.19^a^	8.29 ± 0.32^c^	7.81 ± 0.21b^c^
Hepatosomatic index	2.60 ± 0.10^ab^	2.32 ± 0.08^a^	3.43 ± 0.19^c^	2.91 ± 0.18^b^

Note: Values are represented mean ± standard error of mean from 12 replicates. The superscripts without same letter in the same row indicate significant difference (*p* < 0.05). BBR, berberine; ATB, antibiotics.

### 3.2 Serum biochemical indicators and enzyme activities of largemouth bass

As shown in Table 3, the serum TC and GLU levels in the BBR group were significantly lower than those in the control, ATB, and BBR + ATB groups (*p <* 0.05). Meanwhile, the serum TBA levels in the BBR group were significantly higher than those in the control, ATB, and BBR + ATB groups (*p <* 0.05). The serum TG, LDL-C, GOT, AKP, and ACP levels in the BBR group were non-significantly lower than those in the control group (*p >* 0.05). Compared with those in the ATB and the BBR + ATB groups, the serum LDL-C, GOT, AKP, and ACP levels were significantly lower in the BBR group (*p <* 0.05). The serum GLU, GOT, AKP, and ACP levels in the ATB group were significantly higher than those in the control group (*p <* 0.05). Compared with those in the control group, the serum TBA levels were significantly lower in the ATB group (*p <* 0.05). The serum TC, TG, LDL-C, and HDL-C levels were not significantly different between the ATB and control groups (*p >* 0.05). Compared with those in the control group, the serum GLU and LDL-C levels were significantly higher in the BBR + ATB group (*p <* 0.05). The HDL-C and TBA levels in the BBR + ATB group were significantly lower than those in the control group (*p <* 0.05). The TC, TG, GOT, AKP and ACP levels were not significantly different between the BBR + ATB and control groups (*p >* 0.05).

**TABLE 3:** Serum biochemical and enzymatic indices of largemouth bass.

Groups	Control	BBR	ATB	BBR + ATB
TC (mmol/L)	8.75 ± 0.56^b^	6.13 ± 0.21^a^	10.36 ± 1.16^b^	9.73 ± 0.93^b^
TG (mmol/L)	6.38 ± 0.60^ab^	5.80 ± 0.55^a^	7.68 ± 0.49^b^	7.10 ± 0.39^ab^
GLU (mmol/L)	40.16 ± 1.94^b^	27.27 ± 2.11^a^	53.51 ± 1.63^c^	48.70 ± 1.57^c^
HDL-C (mmol/L)	2.57 ± 0.13^bc^	3.19 ± 0.41^c^	1.96 ± 0.15^ab^	1.67 ± 0.12^a^
LDL-C (mmol/L)	9.55 ± 0.38^ab^	7.50 ± 0.47^a^	12.09 ± 1.64^b^	13.89 ± 0.68^c^
TBA (µmol/L)	9.20 ± 0.34^b^	13.49 ± 0.59^c^	5.64 ± 0.23^a^	6.15 ± 0.52^a^
GOT (U/g protein)	16.44 ± 1.36^ab^	13.54 ± 1.59^a^	26.68 ± 2.08^c^	21.40 ± 1.18^b^
AKP (U/100 ml)	5.61 ± 0.63^ab^	4.37 ± 0.21^a^	7.21 ± 0.52^c^	6.63 ± 0.50^bc^
ACP (U/100 ml)	7.04 ± 0.67^ab^	6.37 ± 0.43^a^	10.13 ± 0.50^c^	8.33 ± 0.52^b^

Note: Values are represented as mean ± standard error of mean from 12 replicates. The superscripts without same letter in the same row indicate significant difference (*p* < 0.05). BBR, berberine; ATB, antibiotics; TC, total cholesterol; TG, triglyceride; GLU, glucose; HDL-C, high-density lipoprotein-cholesterol; LDL-C, low-density lipoprotein-cholesterol; TBA, total bile acids; GOT, glutamic oxalacetic transaminase; AKP, alkaline phosphatase; ACP, acid phosphatase.

### 3.3 Activity of liver-related enzymes in largemouth bass

As shown in Table 4, the hepatic HK, PK, G6P and GOT activities in the BBR group were significantly higher than those in the control, ATB, and BBR + ATB groups (*p <* 0.05). The PEPCK, PFK and PC activities in the BBR group were not significantly different from those in the control group (*p >* 0.05) but were significantly lower than those in the ATB group. Meanwhile, the PEPCK and PC activities in the BBR group were significantly lower than those in the BBR + ATB group. Compared with those in the control group, the PEPCK, PFK and PC activities were significantly higher in the ATB group (*p <* 0.05). However, the HK, PK, G6P and GOT activities were not significantly different between the ATB and control groups (*p >* 0.05). Additionally, the PEPCK, PFK, HK, PK, PC, G6P and GOT activities were not significantly different between the BBR + ATB and control groups (*p* > 0.05).

**TABLE 4:** Activities of metabolism-related enzymes in the liver of largemouth bass.

Groups	Control	BBR	ATB	BBR + ATB
PEPCK (ng/L)	471.40 ± 18.18^ab^	426.24 ± 24.10^a^	750.35 ± 38.16^c^	518.32 ± 16.13^b^
PFK (ng/L)	21.73 ± 2.10^a^	19.42 ± 0.82^a^	26.89 ± 1.37^b^	20.23 ± 1.18^a^
HK (ng/L)	4.41 ± 0.40^a^	5.70 ± 0.17^b^	4.29 ± 0.48^a^	4.39 ± 0.31^a^
PK (ng/L)	30.43 ± 1.96^a^	46.61 ± 4.43^b^	30.18 ± 1.41^a^	32.88 ± 1.70^a^
PC (ng/L)	16.80 ± 0.71^ab^	15.83 ± 0.49^a^	21.11 ± 0.95^c^	18.09 ± 0.50^b^
G6P (ng/L)	26.15 ± 2.27^a^	32.13 ± 1.05^b^	22.54 ± 1.34^a^	25.97 ± 0.71^a^
GOT (ng/L)	2.10 ± 0.21^a^	5.19 ± 1.11^b^	1.53 ± 0.18^a^	1.36 ± 0.17^a^

Note: Values are represented as mean ± standard error of mean from 12 replicates. The superscripts without same letter in the same row indicate significant difference (*p* < 0.05). BBR, berberine; ATB, antibiotics; PEPCK, phosphoenolpyruvate carboxykinase; PFK, phosphofructokinase; HK, hexokinase; PK, pyruvate kinase; PC, pyruvate carboxylase; G6P, glucose-6-phosphatase; GOT, glutamic oxalacetic transaminase.

### 3.4 Analysis of intestinal microbiota α-diversity

High-throughput sequencing analysis revealed at least 53251.33 ± 8208.51 high-quality bacterial 16S rRNA gene reads for each of the 24 samples in the four groups. After clustering with 97% similarity, at least 403.67 ± 187.11 operational taxonomic units were obtained for each group. The coverage index of all samples was higher than 0.99, indicating that the sequences covered almost all types and that the sequencing results were reliable and representative. The α-diversity of intestinal microbiota of each group of samples is shown in Table 5. Compared with that in the control, ATB, and BBR + ATB groups, the Chao one index was higher in the BBR group, indicating that BBR increased species community richness. In contrast, the Shannon and Simpson indices in the ATB and BBR + ATB groups were lower than those in the control group, indicating that BBR decreased community diversity.

**TABLE 5:** Richness and diversity index of bacteria in the samples at 97% similarity level.

Groups	Control	BBR	ATB	BBR + ATB
Chao1	718.66 ± 165.07	908.00 ± 211.50	406.47 ± 75.64	844.03 ± 189.53
Dominance	0.14 ± 0.04^ab^	0.09 ± 0.02^a^	0.29 ± 0.10^ab^	0.33 ± 0.09^b^
Observed otus	713.50 ± 164.08	902.33 ± 209.73	403.67 ± 76.39	842.67 ± 189.80
Pielou e	0.56 ± 0.05^ab^	0.64 ± 0.05^b^	0.47 ± 0.08^ab^	0.43 ± 0.07^a^
Shannon	5.32 ± 0.67	6.19 ± 0.58	4.09 ± 0.72	4.22 ± 0.79
Simpson	0.86 ± 0.04^ab^	0.91 ± 0.02^b^	0.71 ± 0.10^ab^	0.67 ± 0.09^a^
Reads	53251.33 ± 3,351.11	57233.33 ± 4810.38	54559.00 ± 4146.23	65159.33 ± 2815.17

Note: Values are represented as mean ± standard error of mean from six replicates. The superscripts without same letter in the same row indicate significant difference (*p* < 0.05). BBR, berberine; ATB, antibiotics.

### 3.5 Changes in the intestinal microbiota community of largemouth bass

**FIGURE 1:**
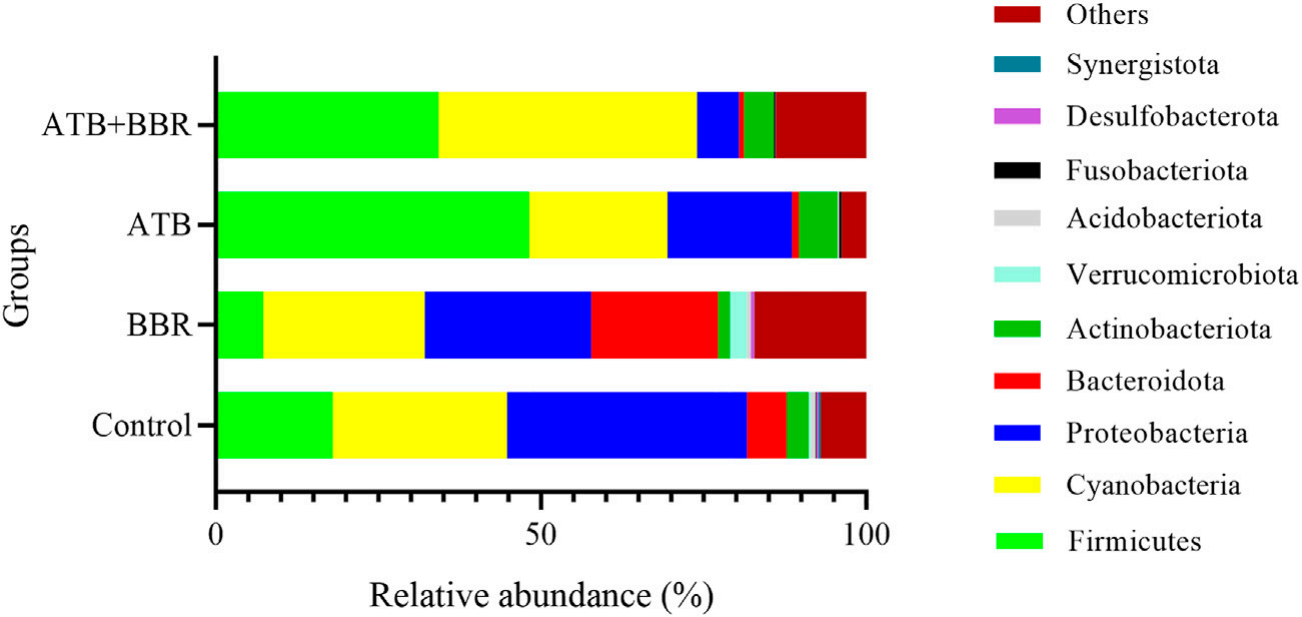
Composition of gut microbiota at the phylum level in largemouth bass. BBR, berberine; ATB, antibiotics.

As shown in Figure 1, at the phylum level, the major microbial composition of each treatment group included Firmicutes, Cyanobacteria, Proteobacteria, Bacteroidota and Actinobacteria. Compared with that in the control group (17.93%), the intestinal Firmicutes content in the BBR group (7.26%) was significantly lower, but was significantly higher in the ATB (48.14%) and ATB + BBR group (34.17%). Compared with that in the control group (6.12%), the Bacteroidota content was significantly higher in the BBR group (19.48%) and significantly lower in the ATB (1.06%) and ATB + BBR groups (0.76%). The Proteobacteria contents in the BBR (25.62%), ATB (19.16%), and ATB + BBR (6.43%) groups were significantly lower than that in the control group (36.85%). The composition of Cyanobacteria was rich in all treatment groups (control: 26.83%; BBR: 24.81%; ATB: 21.29%; ATB + BBR: 39.82%).

**FIGURE 2:**
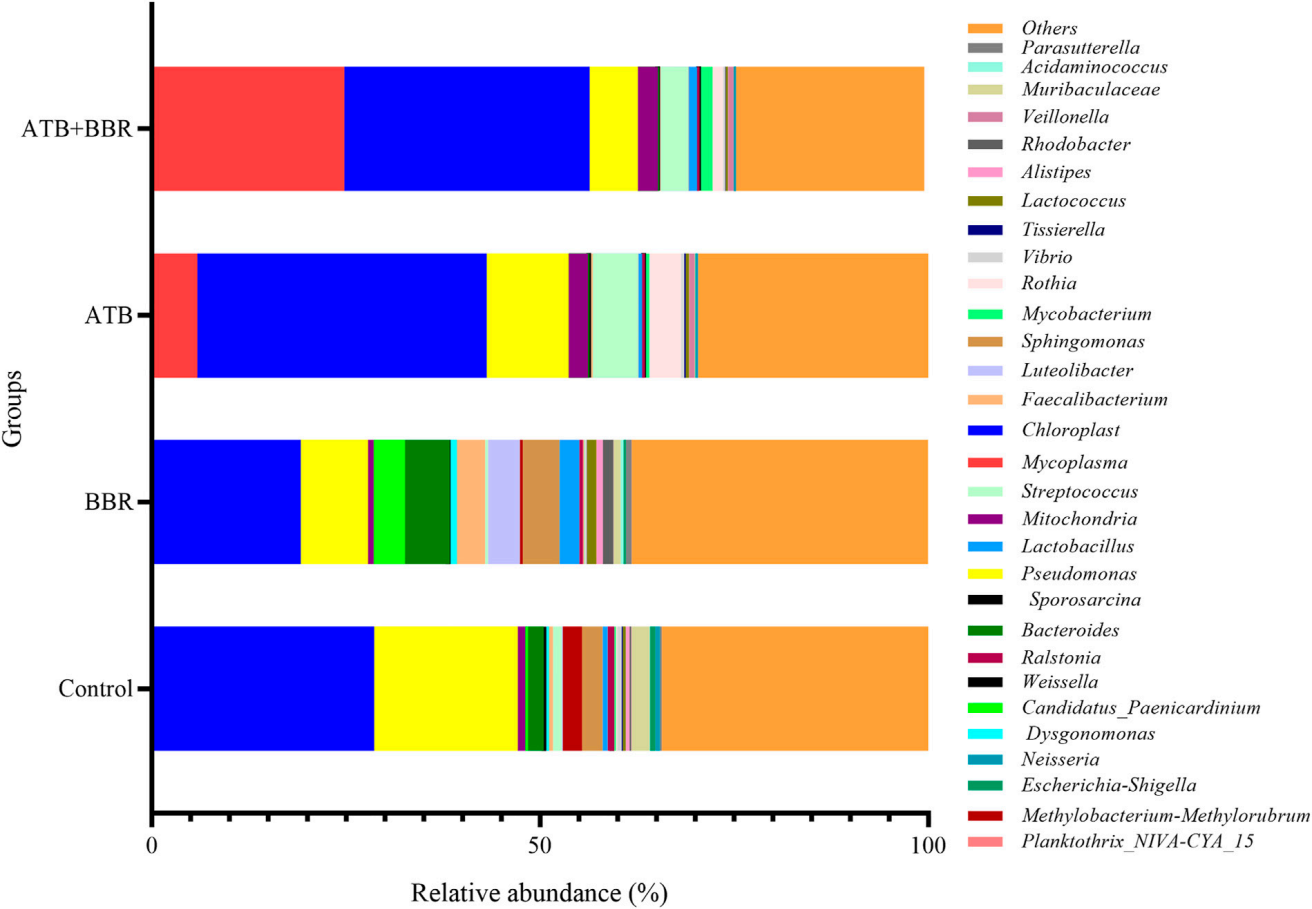
Composition of gut microbiota at the genus level in largemouth bass. BBR, berberine; ATB, antibiotics.

As shown in Figure 2, at the genus level, the *Mycoplasma* content in the ATB (5.85%) and ATB + BBR groups (24.78%) was higher than that in the control group (0.1%). Compared with that in the control group (28.54%), the *Chloroplast* content was lower in the BBR group (19.00%) and higher in the ATB (37.28%) and ATB + BBR groups (31.61%). The *Pseudomonas* content in the BBR (8.69%), ATB (10.55%), and ATB + BBR groups (6.20%) was lower than that in the control group (18.50%). The *Candidatus Paenicardinium* content in the BBR group (1.37%) was higher than that in the control group (0.35%). However, *Candidatus Paenicardinium* was not detected in the ATB and ATB + BBR groups. Compared with that in the control group (2.05%), the *Bacteroides* content was higher in the BBR group (5.80%) and lower in the ATB (0.17%) and ATB + BBR groups (0.16%). The contents of *Faecalibacterium*, *Lactobacillus*, and *Lactococcus* in the control group were 0.53%, 0.65%, and 0.39%, respectively, while those in the BBR group were 3.62%, 2.56%, and 1.23%, respectively. The *Streptococcus* content in the BBR group (0.42%) was lower than that in the control (1.14%), ATB (5.84%), and ATB + BBR groups (3.63%).

**FIGURE 3:**
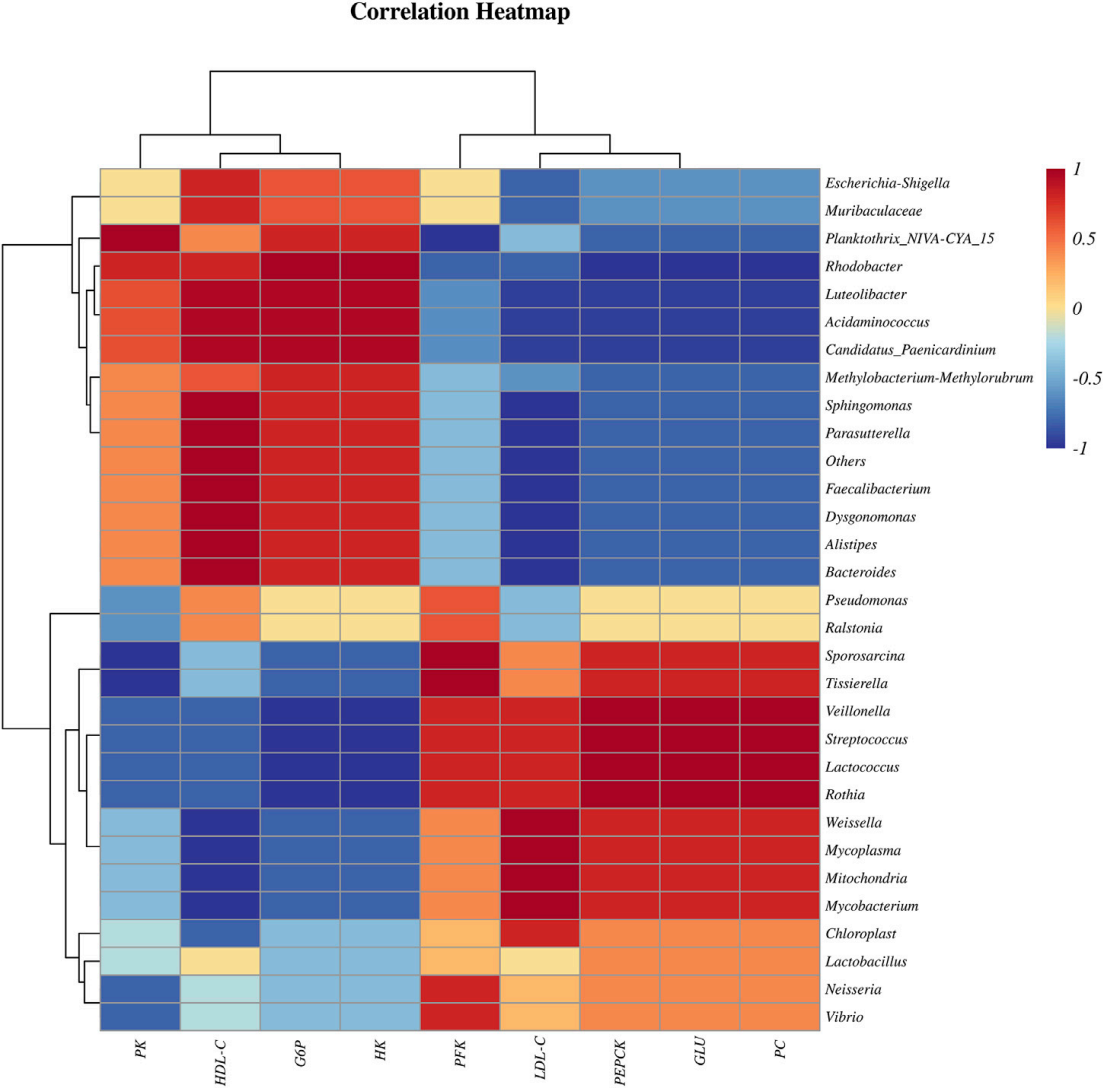
Spearman correlation coefficient heatmap of bacteria and physiological and biochemical indexes.

The Spearman correlation coefficient of bacteria and physiological and biochemical indexes indicated that, at genus level, the *Pseudomonas*, *Candidatus Paenicardinium*, *Bacteroides* and *Faecalibacterium* were positively correlated with liver glycolytic rate-limiting enzymes (PK, G6P, HK) and serum HDL-C; *Mycoplasma*, *Chloroplast*, *Streptococcus*, *Lactobacillus* and *Lactococcus* are positively correlated with the liver rate limiting enzymes (PFK, PEPCK, PC) related to gluconeogenesis, serum LDL-C and GLU (Figure 3).

### 3.6 Histomorphological observations of the liver of largemouth bass

**FIGURE 4:**
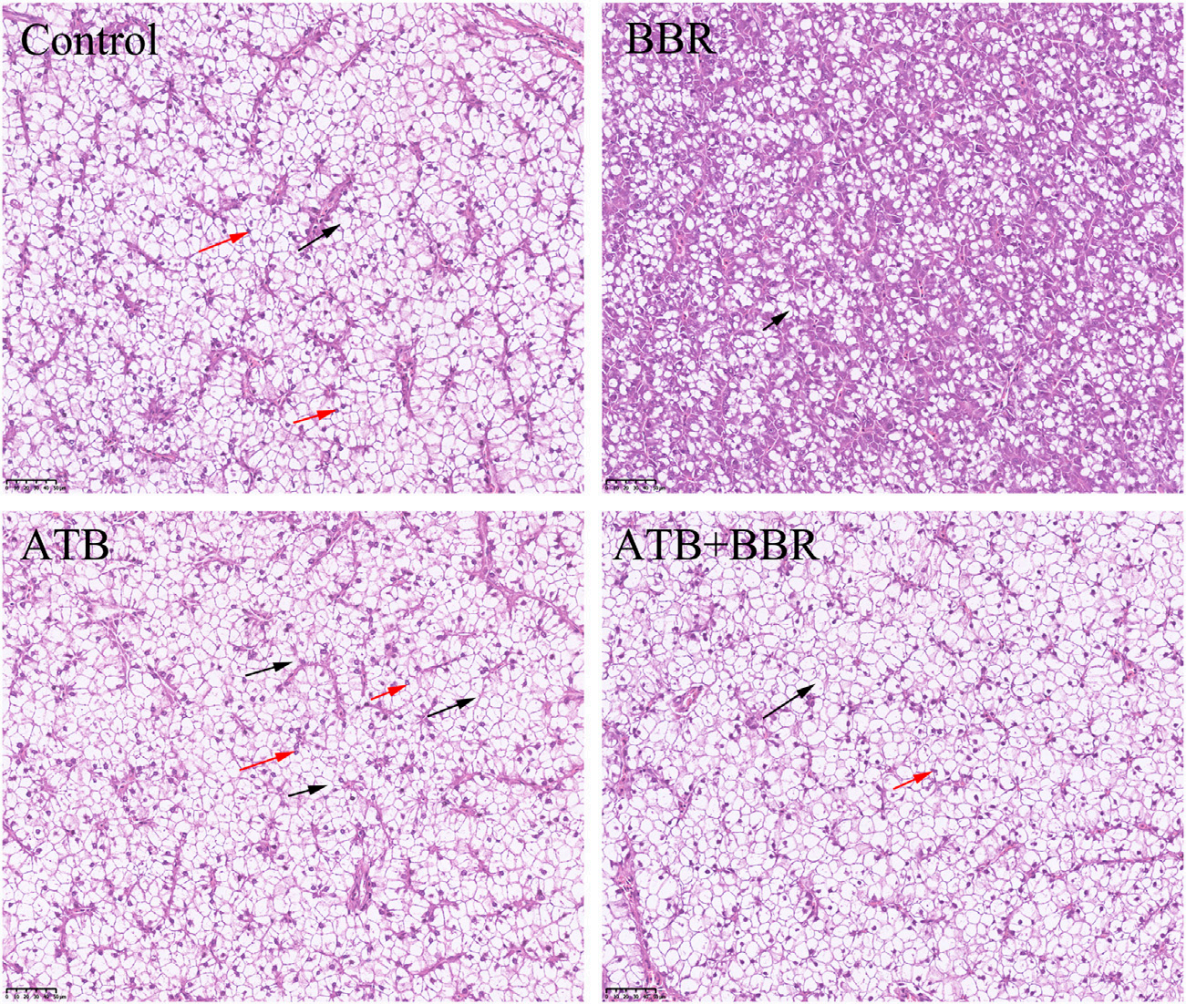
Hematoxylin and eosin staining of largemouth bass liver (×10). The red arrows in the picture represent the nucleus pushed to the edge of the cell; the black arrows represent the disappearance of the cell nucleus and cytoplasmic vacuolization. BBR, berberine; ATB, antibiotics.

**FIGURE 5:**
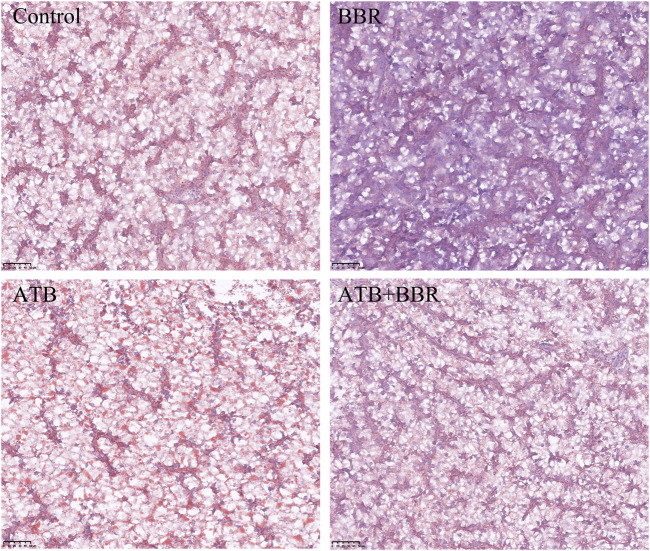
Oil red staining of largemouth bass liver (×10). The red colour in the picture represents lipid drops. BBR, berberine; ATB, antibiotics.

As shown in Figure 4, HE staining analysis of largemouth bass liver revealed that compared with those in the BBR group, the hepatocytes exhibited larger size, increased vacuolization, and enhanced number of nuclei at the edge in the control, ATB, and ATB + BBR groups. Oil Red O staining of liver revealed increased lipid distribution in the liver tissue of the control, ATB, and ATB + BBR groups (Figure 5).

### 3.7 Results of *in-vitro* culture of intestinal microbiota

**FIGURE 6:**
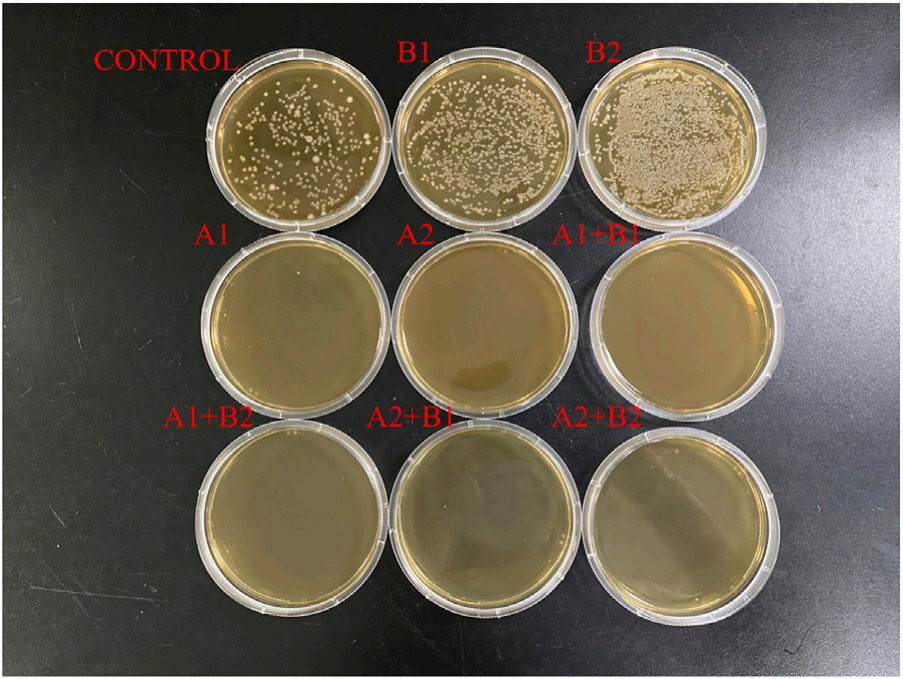
*In-vitro* culture of gut microbiota of largemouth bass. CONTROL: control; B1: low concentration of berberine; B2: high concentration of berberine; A1: low concentration of mixed antibiotics; A2: high concentration of mixed antibiotics.

The isolated culture of intestinal microbiota of largemouth bass is shown in Figure 6. The number of intestinal flora in the control, B1, and B2 groups was 4.48 × 10^8^, 1.93 × 10^9^, and 2.28 × 10^9^ CFU, respectively. Compared with that in the control group, the number of intestinal microbiota significantly increased in the BBR group (low concentration B1, high concentration B2) and decreased in the mixed ATB group (low concentration A1, high concentration A2). The number of intestinal microbiota decreased significantly and was almost 0. Additionally, the number of intestinal microbes was almost 0 in groups treated with both mixed ATB and BBR (A1 + B1, A1 + B2, A2 + B1, and A2 + B2).

The single colony sequencing results revealed that the characteristic bacteria in the control group were *Bacillus sp.*, *Aeromonas hydrophila*, and *Citrobacter freundii*. The dominant characteristic bacteria in the B group were *Enterobacter cloacae*.

As shown in Table 6, the API test results of the characteristic strains of group B were as follows: positive reactions with arginine (ADH), ornithine (ODC), citric acid (CIT), pyruvate (VP), GLU, rhamnose (RHA), sucrose (SAC), mydrose (MEL), and arabinose (ARA); negative reactions with tryptophan deamination, gelatin, and cytochrome oxidase (OX) reactions.

**TABLE 6:** API test results of Group B characteristic strains.

Test	Active ingredient	Reactions/Enzymes	Result
ONPG	o-Nitrophenyl-galactoside	beta-galactosidase	Positive
ADH	arginine	Arginine dihydrolase	Positive
LDC	lysine (Lys), an essential amino acid	lysine decarboxylase	Negative
ODC	ornithine (Ornithine), an essential amino acid	Ornithine decarboxylation	Positive
CIT	sodium citrate	Citric acid utilization	Positive
H2S	sodium thiosulfate Na_2_ S_2_ O_3_	H_2_ S generated	Negative
URE	urea (NH2)2CO	urease (enzyme)	Negative
TDA	tryptophan (Trp), an essential amino acid	tryptophan deaminase (TSLD)	Negative
IND	tryptophan (Trp), an essential amino acid	indole production	Negative
VP	pyruvate	3-Hydroxybutanone produces acetylmethylmethanol	Positive
GEL	Kohn Gelatin	gelatinase	Negative
GLU	glucose	Fermentation/oxidation	Positive
MAN	mannitol	Fermentation/oxidation	Positive
INO	inositol	Fermentation/oxidation	Weakly positive
SOR	sorbitol C6H14O6 (sugar substitute and mild laxative)	Fermentation/oxidation	Positive
RHA	rhamnose	Fermentation/oxidation	Positive
SAC	fructose	Fermentation/oxidation	Positive
MEL	disaccharide	Fermentation/oxidation	Positive
AMY	amygdalin	Fermentation/oxidation	Positive
ARA	arabinose (type of sugar)	Fermentation/oxidation	Positive
OX	Tetramethyl-p-phenylenediamine vitamin C	cytochrome oxidase	Negative

## 4 Discussion

In this study, the supplementation of BBR improved the growth of largemouth bass and decreased the viscera weight and hepatosomatic indices. However, the supplementation of ATB exerted contrasting effects, and the effect of ATB + BBR was similar to that of ATB alone. A previous study (Zhang et al., 2013) demonstrated that the moderate addition of BBR improved the growth of largemouth bass, which can be attributed to the ability of BBR to regulate GLU metabolism in largemouth bass and promote intestinal health. Additionally, the supplementation of ATB can adversely affect growth and gut health (Limbu et al., 2018; Sun et al., 2020) and reduce the diversity of gut microbial community composition (Ramirez et al., 2020). In this study, when ATB and BBR were simultaneously added, the BBR could not mitigate the adverse effects of ATB on largemouth bass. This indicates that the use of ATB may disrupt the key pathway through which BBR exerts its regulatory effects. Further studies are needed to elucidate the exact mechanisms.

GOT and GPT are commonly known as transaminases, which are indicators of liver function and are mainly expressed in hepatocytes. Hepatocyte damage induced by inflammation, necrosis, and toxicity promotes the release of GOT and GPT into the blood (Wróblewski 1958). Compared with those in the control group, the serum GOT level were significantly downregulated in the BBR group and significantly upregulated in the ATB group at the end of the experimental period. This indicates that the use of ATB promoted liver damage in largemouth bass. BBR effectively regulated the health of the liver. HE and Oil Red O staining of liver tissues revealed that hepatocytes in the BBR group were small with decreased vacuolisation, centred nuclei, and decreased lipid distribution in the liver tissue. The liver was evaluated by paying special attention to hypertrophy and nuclei position of the hepatocytes (Betancor et al., 2017). Previous studies indicated that feeds high-carbohydrates levels caused liver damage of fish, which were manifested in increased vacuolization and movement of nuclei to the margins (Magalheãs et al., 2021; Zhao et al., 2022). This suggests that the use of BBR is beneficial to the liver health of largemouth bass and that antibiotics are detrimental to liver health. These findings are consistent with those of Björnsson and Serranti et al., who reported the adverse effects of ATB on the liver (Serranti et al., 2013; Björnsson 2017).

Enzymes are specific and catalyze specific reactions, the increase or decrease of enzyme activity, which we believe may indirectly prove that this reaction is promoted or inhibited (Koutedakis et al., 1993; Mahboob et al., 2005), and the change of GLU level further proves that enzyme activities may be appropriate. The serum GLU, TC, TG, HDL-C, and LDL-C levels are key indicators of glucolipid metabolism (Turnbaugh et al., 2006; Luo et al., 2017). The studies demonstrated that GLU metabolism is closely related and complementary to lipid metabolism and positively correlated with adiposity, fasting insulin, and TG and negatively correlated with HDL-C (Lawlor et al., 2012; Khan et al., 2018; Dahal et al., 2022). Compared with those in the control group, the serum TC, TG, and LDL-C levels were downregulated and the serum TBA and HDL-C levels were significantly upregulated in the BBR group. This may indicate that BBR positively regulated glucolipid metabolism in largemouth bass. The TC, TG, LDL-C, and HDL-C levels were not significantly different between the ATB and control groups. The serum TBA level in the ATB group were significantly lower than those in the control group, indicating that the addition of ATB may not promote serum GLU metabolism and lipid metabolism in largemouth bass. In contrast, the simultaneous addition of ATB and BBR significantly decreased the HDL-C and TBA levels but did not affect the TC, TG, GOT, AKP, and ACP levels in largemouth bass. So it is possible to draw a conclusion that the addition of ATB suppressed the positive regulatory effects of BBR on glucolipid metabolism. This further demonstrated that the use of ATB inhibited the key pathway involved in the regulatory effects of BBR.

The serum GLU level in the BBR group were significantly lower than those in the control group, indicating that the addition of BBR decreased the blood GLU level in largemouth bass, which is consistent with the results of Pan et al. (2019). The hepatic activities of HK, PK, and G6P, which are the three key enzymes of glycolysis, in the BBR group were significantly higher than those in the control group. This may indicate that the addition of BBR promoted glycolysis in largemouth bass, which is consistent with the suppressive effects of BBR on blood GLU levels in largemouth bass. The serum GLU level in the ATB group were significantly higher than those in the control group. The HK, PK, and G6P activities were not significantly different between the ATB and control groups. Previous studies have reported that ATB upregulates blood GLU in the organism, which may be associated with a reduction in the overall diversity of the intestinal microbial community (Carvalho et al., 2012; Vrieze et al., 2014; Zarrinpar et al., 2018). This is consistent with the lack of changes in the levels of TC, TG, LDL-C, and HDL-C in largemouth bass after the addition of ATB. This study most likely demonstrated that the addition of both ATB and BBR significantly increased blood GLU level in largemouth bass but did not significantly affect the HK, PK, and G6P activities when compared with the control. The use of ATB may have disrupted key targets of BBR, which may be related to the community diversity of intestinal microbes.

The addition of ATB disrupted the intestinal microbial community, decreased bacterial diversity, and induced dysbiosis of the intestinal flora (Modi et al., 2014; Fröhlich et al., 2016). Compared with that in the control group, the intestinal microbial community richness was upregulated in the BBR group and the community diversity was downregulated in the ATB and BBR + ATB groups. These results were consistent with those of Modi et al. (2014) and Fröhlich et al. (2016). The findings of the ATB + BBR group indicated that BBR did not mitigate the adverse effects of ATB. Previous studies demonstrated that the role of BBR in regulating host glycolipid metabolism is related to the interaction between BBR and intestinal microflora (Chen et al., 2022; Gong et al., 2022). By changing the diversity and composition of intestinal microflora and then changing its metabolites, which could further achieve the goal of regulating downstream key targets and signaling pathways (for example the *FXR-FGF 15/19*, *P-AKT SER473*,*GLP-1* and insulin secretion, *etc.*), the BBR ultimately improved host metabolism (He et al., 2022). The bactericidal action of ATB in this research destroyed the main pathway (the intestinal microbiota) that BBR to play the role of reducing fish serum glucose and improving liver health. The above conclusions were further confirmed by the *in-vitro* test, which revealed that the addition of BBR significantly increased the number of intestinal culturable microbiota, but ATB decreased it, and the addition of both BBR and ATB at the same time still inhibited the growth of intestinal microbiota seriously.

At the phylum level, the intestinal Firmicutes contents were significantly downregulated in the BBR group but upregulated in the ATB and ATB + BBR groups. Meanwhile, the Bacteroidota content was significantly upregulated in the BBR group and downregulated in the ATB and ATB + BBR groups. The intestinal Firmicutes/Bacteroidota relative ratio was downregulated in the BBR group and upregulated in the ATB and ATB + BBR groups. High proportions of Firmicutes/Bacteroidota are associated with a carbohydrate diet (Fukuda et al., 2011). Firmicutes/Bacteroidota are often used as indicators to measure obesity status (Li et al., 2015; Rizzatti et al., 2017). The supplementation of BBR mitigated “obesity” in largemouth bass, whereas ATB had no effect. Furthermore, the inability of BBR to function in the presence of ATB is consistent with the results of gut microbial community diversity analysis. This suggests that the supplementation of ATB suppressed the positive regulatory effects of BBR in glycolipid metabolism in largemouth bass. Furthermore, the use of ATB may inhibit a key pathway, that is, to reduce the gut microbial community diversity, through which BBR exerts its regulatory role.

Proteobacteria are gram-negative bacteria and a major clade of bacteria that include several pathogenic bacteria, such as *Escherichia coli, Salmonella, Vibrio cholerae, Helicobacter pylori*, and other well-known species. In mammals, intestinal dysbiosis is usually accompanied by increased contents of Proteobacteria. In some intestinal settings, the content of Proteobacteria is a potential microbial signature of intestinal disease and inflammation and an identification criterion for intestinal health (Kholil et al., 2015; Rizzatti et al., 2017). Proteobacteria content was significantly downregulated in the BBR, ATB, and ATB + BBR groups. This indicated that the supplementation of BBR and ATB decreased the relative abundance of enteropathogenic bacteria. At the genus level, the *Pseudomonas* content was downregulated in the BBR, ATB, and ATB + BBR groups. Several species in the genus *Pseudomonas* are fish pathogenic bacteria (Altinok et al., 2006; Wiklund 2016), such as *Pseudomonas fluorescens* and *Pseudomonas aeruginosa*, which are considered pathogenic microorganisms in aquaculture (Bodey et al., 1983; Miquel et al., 2013). The results of this study suggest that the supplementation of BBR and ATB decreased the relative abundance of pathogenic bacteria in the gut flora. The BBR group exhibited increased levels of *Faecalibacterium*, *Lactobacillus*, and *Lactococcus. Faecalibacterium*, a major member of the intestinal microbiota in healthy humans, produces butyric acid, which exerts anti-inflammatory effects and can alleviate obesity in humans. The downregulation of *Faecalibacterium* promotes an inflammatory response (Reid and Burton 2002; Ferreira-Halder et al., 2017; Lopez-Siles et al., 2017). Previous researches indicated that *Faecalibacterium* as probiotic improved the immunity and changed intestinal microbiota composition of shrimp and turbot (Guo et al., 2022; Butucel et al., 2023) The supplementation of BBR increased the contents of *Faecalibacterium*. This suggests that *Faecalibacterium* promotes intestinal health in largemouth bass, reduces the associated inflammatory response, and regulates body metabolism. *Lactobacillus* prevents intestinal infections. The incorporation of *Lactobacillus* strains ingested into the intestine modulates the vaginal flora to a healthy state (van Hylckama Vlieg et al., 2006; Petrova et al., 2017). *Lactococcus lactis* is a globular gram-positive anaerobic bacterium widely used in the production of fermented dairy products and occupies a key position in the manufacturing of fermented foods with beneficial effects on human health (van de Guchte et al., 1992; Modi et al., 2014). *Lactobacillus* was also proved to be a kind of probiotics commonly used in aquatic production, which can improve the growth performance, regulate intestinal health, and improve disease resistance of aquatic animals (Suzer et al., 2008; Ramachandran et al., 2014). These results indicate that the use of BBR reduces the relative abundance of pathogenic bacteria in the intestinal flora and increases the relative abundance of beneficial bacteria, promoting intestinal health. Meanwhile, the use of ATB kills harmful bacteria and beneficial bacteria in the intestinal tract. Thus, the use of BBR for the treatment of bacterial diseases in aquatic animals will achieve twice the result with half the effort when compared with the ATB.

The results of the *in-vitro* culture assays with intestinal microbiota further validated the direct effect of BBR on intestinal microbiota composition. In the group treated with BBR, the dominant characteristic bacteria was *E. cloacae*, and this bacteria also was found (only <0.1%) within molecular methods *in vivo*. The difference of the above results are due to the advantages and limitations of each method, and combination of the two methods reflected the actual situation more comprehensively (Kellenberger, 2001; Di Bella et al., 2013). And this strain reacted positively with arginine, ornithine, citrate, and pyruvate, which are important intermediate metabolites involved in glycolysis, tricarboxylic acid cycle and ornithine cycle (Benuck et al., 1971; Akram 2014; Zangari et al., 2020). The positive reactions to GLU, RHA, SAC, MEL, and ARA indicated the carbohydrate metabolism ability of the characteristic bacteria obtained from the BBR group.

## 5 Conclusion

The results of this study demonstrated that the supplementation of BBR decreased the blood GLU level and improved GLU metabolism in largemouth bass. Comparative analysis of the results of experiments with ATB supplementation revealed that BBR regulated GLU metabolism in largemouth bass might be attributable to the modulation of BBR on intestinal microbiota.

## Data Availability

The datasets presented in this study can be found in online repositories. The names of the repository/repositories and accession number(s) can be found below: https://www.ncbi.nlm.nih.gov/sra/PRJNA931008.
